# Putting Together Pieces of the Lateral Septum: Multifaceted Functions and Its Neural Pathways

**DOI:** 10.1523/ENEURO.0315-21.2021

**Published:** 2021-12-02

**Authors:** Candace A. Rizzi-Wise, Dong V. Wang

**Affiliations:** Department of Neurobiology and Anatomy, Drexel University College of Medicine, Philadelphia, PA 19129

**Keywords:** anxiety, fear, feeding, hippocampus, lateral septum, memory

## Abstract

The lateral septum (LS) is implicated as a hub that regulates a variety of affects, such as reward, feeding, anxiety, fear, sociability, and memory. However, it remains unclear how the LS, previously treated as a structure of homogeneity, exhibits such multifaceted functions. Emerging evidence suggests that different functions of the LS are mediated largely by its diverse input and output connections. It has also become clear that the LS is a heterogeneous region, where its dorsal and ventral poles play dissociable and often opposing roles. This functional heterogeneity can often be explained by distinct dorsal and ventral hippocampal inputs along the LS dorsoventral axis, as well as antagonizing connections between LS subregions. Similarly, outputs from LS subregions to respective downstream targets, such as hypothalamic, preoptic, and tegmental areas, also account for this functional heterogeneity. In this review, we provide an updated perspective on LS subregion classification, connectivity, and functions. We also identify key questions that have yet to be addressed in the field.

## Significance Statement

The lateral septum (LS) is a major relay that connects the hippocampus with various subcortical regions; however, how the LS communicates with these regions and processes relevant information has not been well studied. The past several years has brought a number of publications using multidisciplinary approaches, including optogenetics, electrophysiology, and calcium imaging, to elucidate the neural circuitry and functions of the LS. Here, we summarize and integrate current knowledge about the LS circuitry to inspire further research. We propose that the multifaceted functions of the LS are mainly mediated by its diverse input and output connections, and that LS subregions often antagonize each other in competition for controlling behavioral outputs.

## Introduction

The lateral septum (LS) is a major site that connects the hippocampus with multiple subcortical regions, including the lateral hypothalamic area (LHA), lateral preoptic area (LPO), medial hypothalamic area (MHA), medial preoptic area (MPO), ventral tegmental area (VTA), supramammillary area (SUM), medial septum (MS), nucleus accumbens, periaqueductal gray, etc. (Risold and Swanson, 1997b; Sheehan et al., 2004). Expectedly, this central position enables the LS to integrate a variety of emotional, spatial, and cognitive information for regulating behavioral outputs. Pioneer work investigating the LS revealed two of its important roles: reward and “septal rage”. The role in reward is readily evident as animals quickly learn to self-stimulate the LS and continue to do so until they are physically exhausted (Olds and Milner, 1954; Prado-Alcala et al., 1984). Septal rage refers to a series of exaggerated emotional and defensive responses to nonthreatening stimuli after damage to the LS (Brady and Nauta, 1953). Since then, accumulating evidence has evolved to position the LS as a critical structure for many other functions beyond reward and rage.

The LS is composed of predominantly GABAergic neurons, ranging from 85% to 100% based on different estimations (Risold and Swanson, 1997a; Zhao et al., 2013; Wong et al., 2016). Correspondingly, most LS neurons express molecular markers for GABAergic neurons: subsets express somatostatin (SST), calbindin or calretinin, but very few express parvalbumin (Zhao et al., 2013; Carus-Cadavieco et al., 2017; Besnard et al., 2019, 2020). The LS is also enriched with various neuropeptide receptors, including vasopressin, oxytocin, ghrelin, glucagon-like peptide 1 (GLP-1), corticotropin-releasing factor (CRF), neuropeptide Y, and neurotensin receptors (Risold and Swanson, 1997a; Kask et al., 2001; Anthony et al., 2014; Azevedo et al., 2020). Different LS subpopulations appear to express distinct combinations of these neuropeptide markers and, thus, exhibit distinct physiological functions (Risold and Swanson, 1997a; Zhao et al., 2013; Besnard et al., 2019, 2020).

**Figure 1.**
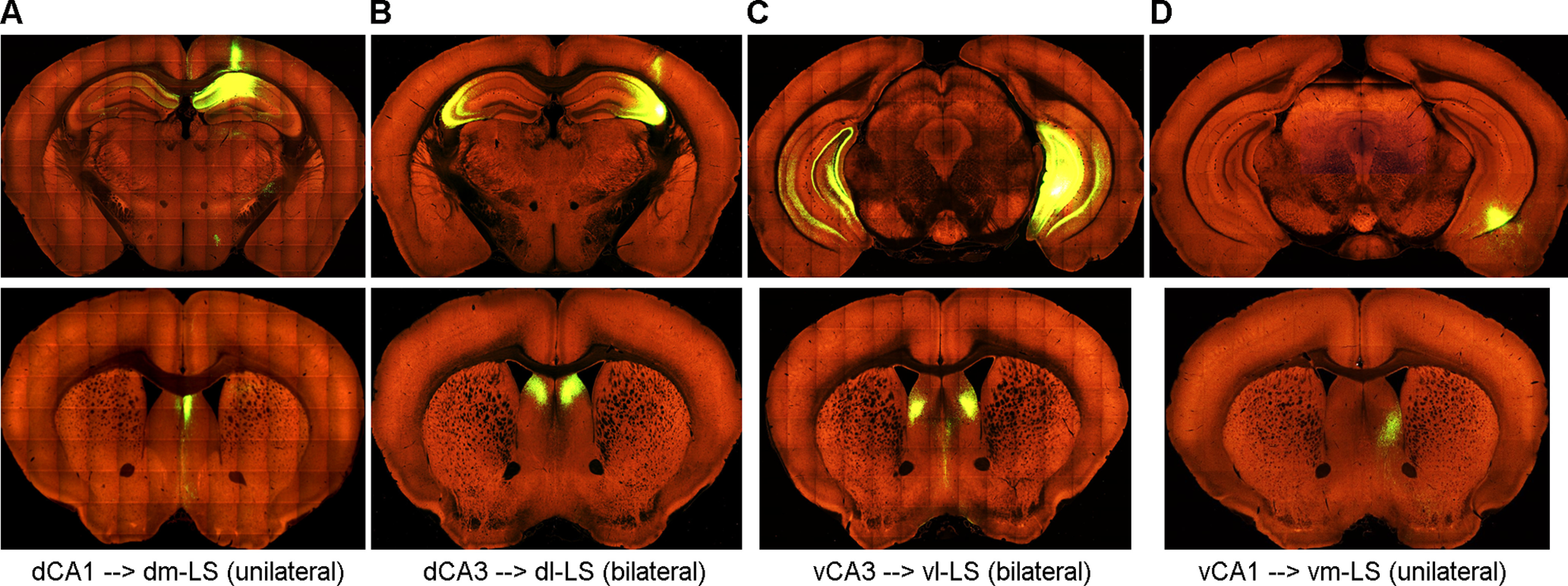
Subregion specific hippocampus→LS projections. ***A–D***, top, Coronal brain sections from four individual mice showing unilateral injection (right hemisphere) of AAV-Syn-GFP in the dCA1 (***A***), dCA3 (***B***), vCA3/DG (***C***), and vCA1 (***D***), respectively. Bottom, Coronal brain sections from the same four mice showing the projection in the dm, dl, vl, and vm subregions of the LS, respectively. All brain section photographs are adapted from *Allen Brain Atlas – Mouse Connectivity* (Oh et al., 2014).

**Figure 2.**
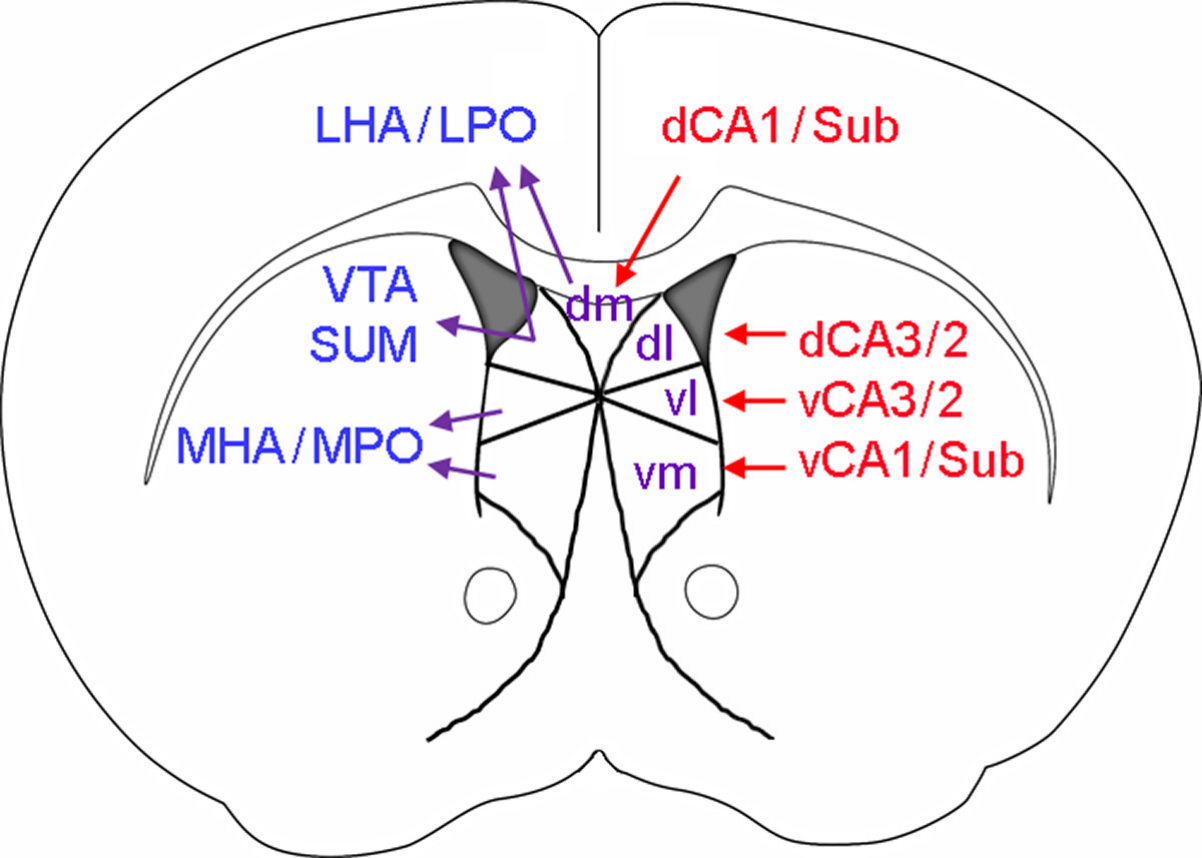
Major LS connections. LS subregions (dm, dl, vl, and vm) and their connections with hippocampal, hypothalamic, and other major areas. CA3/2, CA3 and CA2; d/v, dorsal/ventral; LHA, lateral hypothalamic area; LPO, lateral preoptic area; MHA, medial hypothalamic area; MPO, medial preoptic area; Sub, subiculum; SUM, supramammillary nucleus; VTA, ventral tegmental area.

The multifaceted functions of the LS are also a direct result of its diverse input/output connections (Risold and Swanson, 1997b; Oh et al., 2014; Tsanov, 2018). The LS receives the densest input from the hippocampus (Sheehan et al., 2004), and accordingly, we attempted to divide the LS into four subregions solely based on their inputs from different hippocampal subregions. These include the dorsomedial (dm), dorsolateral (dl), ventrolateral (vl), and ventromedial (vm) LS, which preferably receive inputs from hippocampal dCA1/Sub, dCA3, vCA3, and vCA1/Sub, respectively (Figs. 1, 2). Notably, while the LS receives a unilateral projection from the CA1 (Fig. 1*A*,*D*), it receives bilateral projections from the CA3 (Fig. 1*B*,*C*). Similarly, outputs from these LS subregions preferentially target distinct subcortical regions (Fig. 2): the dm-LS mainly projects to the LHA and LPO; the dl-LS projects to the LHA, LPO, VTA and SUM; and the vl-LS and vm-LS project to the MHA and MPO. On the other hand, there is only sparse projection between the LS and neocortical regions (Risold and Swanson, 1997b; Sheehan et al., 2004). The collection of these works uses a combination of traditional tracing methods and more modern use of viral tracing. While this allows for a solid foundation to build a visualization of comprehensive circuitry, we acknowledge that there are still pathways emerging with the continued study of the LS, particularly using viral tracing techniques, that will need further characterization (Carus-Cadavieco et al., 2017; Jonsson et al., 2017; Chen et al., 2021).

This expansive LS circuitry indicates important subregional differences within the LS, corresponding to diverse or even opposing functions. The opposing function is also supported by findings that LS subregions can directly inhibit each other (Sheehan et al., 2004; Leroy et al., 2018; Oliveira et al., 2021). During our review of the literature, we were able to differentiate studies that specifically target one of the above four LS subregions; however, others likely target two or more of these subregions. To simplify, we refer to dm-LS and dl-LS collectively as dorsal LS (dLS), vl-LS and vm-LS collectively as ventral LS (vLS), and dl-LS and vl-LS collectively as lateral LS. In cases where three or four subregions are targeted, we refer to them collectively as LS. Only recently, modern tools used for circuitry dissections, such as optogenetics and chemogenetics, have been employed to investigate the diverse functions of LS subregions (Yizhar et al., 2011; Roth, 2016). This review will provide a comprehensive overview of the most recent developments in the knowledge of LS circuitry and function, mainly in rodent models, with a specific focus on reward, feeding, anxiety, fear, sociability, and memory.

## LS-Associated Circuitry in Reward

Findings from self-stimulation studies in rodents provided one of the original deductions for the LS having rewarding and reinforcing properties. Only recently has this reward-related function been revisited in the context of LS-associated circuitry involving upstream and downstream connections. Most commonly, experiments studying reward-based behaviors use optogenetic operant conditioning or pharmacological manipulations within cocaine or alcohol use paradigms. These studies indicate that the LS itself can elicit rewarding properties as well as being involved in drug addiction behaviors.

### LS and reward

The pioneering work on the LS demonstrated a repetitive self-stimulation of this region indicative of its role in reward (Olds and Milner, 1954; Prado-Alcala et al., 1984). Building on these early findings, research has hypothesized a connection from the LS to several regions that have been well studied in reward processing, including the VTA, nucleus accumbens, and lateral hypothalamus (Risold and Swanson, 1997b; Sheehan et al., 2004). In support of this, recent studies revealed that pharmacological stimulation of the LS increases VTA dopamine neuronal activity; whereas pharmacological blockade of LS activity decreases dopamine release in the accumbens, indicating a septal control of the VTA in mediating reward (Luo et al., 2011; Jonsson et al., 2017; Vega-Quiroga et al., 2018). Moreover, the lateral LS could directly control the accumbens, a major VTA downstream target, in mediating reward (Zahm et al., 2013; Jonsson et al., 2017). Additionally, a vLS→hypothalamic tuberal nucleus (Tu) pathway has been shown to mediate reward as optogenetic activation of this pathway induces real-time place preference (Mu et al., 2020). Together, three parallel LS pathways that engage well-studied rewarding brain areas, namely, dl-LS→VTA, vLS→Tu, and lateral LS→accumbens pathways, have been implicated in mediating reward.

### LS and drug abuse

Given that the LS projects directly to the VTA and regulates dopamine activity, it is conceivable that the LS is involved in drug seeking and abuse behaviors. Studies quantifying neuronal activation revealed an increase of immediate early gene expression in the LS after injection or context-induced reinstatement of cocaine (Franklin and Druhan, 2000; Zahm et al., 2010; McGlinchey and Aston-Jones, 2018). In addition, many LS molecular markers, such as the GLP-1 receptor and cocaine- and amphetamine-regulated transcript peptide, are subject to change after drug intake (Janzsó et al., 2010; Harasta et al., 2015). These changes are indicative that the LS adapts to drug intake and may subsequently affect drug-seeking behaviors. On the other hand, pharmacological inhibition of the LS by baclofen-muscimol injection reduces motivation to indulge in drug-seeking behavior (Pantazis and Aston-Jones, 2020). Additionally, intra-LS injections of opioid receptor antagonists block behavioral sensitization to morphine (Li et al., 2021). These findings provide compelling evidence of the LS’s role in drug seeking behaviors.

Upstream of the LS, the dorsal hippocampus likely provides contextual information to the dLS for mediating drug seeking behaviors. Corroborating this notion, recent studies revealed a tri-synaptic pathway from the dCA3→dl-LS→VTA that promotes cocaine seeking (Jiang et al., 2018; Luo et al., 2011). Conversely, chemogenetic inhibition of the dCA3→dl-LS pathway attenuates cocaine seeking (McGlinchey and Aston-Jones, 2018; Werner et al., 2020). Additional evidence suggests that the dLS also recruits downstream LHA to regulate cocaine seeking behaviors (Sartor and Aston-Jones, 2012). On the other hand, the role of the ventral hippocampus→vLS pathway in drug abuse remains unknown. It is possible that this ventral pathway indirectly regulates drug seeking behavior, given that the ventral hippocampus plays a critical role in stress and anxiety, and that such negative emotions increase risk of drug seeking and relapse (Sinha, 2001; Fanselow and Dong, 2010). In summary, two dLS-associated pathways, namely, dCA3→dl-LS→VTA and dLS→LHA pathways, have been implicated in drug seeking and abuse. Besides processing drug rewards, the LS is also involved in processing natural rewards such as those associated with feeding, which is discussed below.

## LS-Associated Circuitry in Feeding

The LS has long been thought to play a role in feeding given its dense projection to the hypothalamus, a central site that controls appetite and satiety (Risold and Swanson, 1997b). This notion is also supported by the LS’s role in reward and emotion, often linking food consumption with eating disorders. Feeding is a complex process that involves psychological, physiological, and behavioral mechanisms. Indeed, available evidence supports the LS’s involvement in multiple aspects of feeding behaviors, including food motivation, food intake, and taste preference.

### LS and food motivation

An important aspect of feeding behavior is food motivation, which reflects an effort of food seeking but not necessarily food intake. Food motivation is often studied using operant responding paradigms, in which increased responding (e.g., lever press or nose poke) is interpreted as high motivation for food. Studies that have examined food motivation focus on the function of several receptors in the LS, particularly the GLP-1 and ghrelin receptors, both of which are critical in controlling feeding behavior (Steinert et al., 2017). These receptors appear to exhibit opposite effects: intra-dLS administrations of GLP-1 and its antagonist suppress and increase operant responding for food, respectively (Terrill et al., 2016, 2019), whereas an intra-dLS administration of ghrelin increases operant responding for food (Terrill et al., 2018). Notably, no effect was observed when the same GLP-1 manipulations targeted the intermediate regions of the LS (Terrill et al., 2016). This supports the notion that any role in food motivation is mainly, if not exclusively, confined to the dLS. In addition, the SST-positive neurons in the LS have been shown to drive food motivation (Carus-Cadavieco et al., 2017). Whether these SST-positive and ghrelin (or GLP-1R) neurons are overlapping or exclusive populations within the LS has yet to be investigated. At the circuit level, optogenetic activation (at γ frequency) and inhibition of the infralimbic cortex (IL)→LS pathway increases and decreases food motivation, respectively (Carus-Cadavieco et al., 2017). Overall, the role of the LS in food motivation is in line with the general role of the LS in driving reward and motivation (Olds and Milner, 1954).

### LS and food intake

Separate from the motivation component of feeding, the LS can also directly regulate food intake through pharmacological manipulation of different receptors. Ghrelin, μ-opioid, and GLP-1 receptors all influence food intake: activation of the former two receptors promotes food intake, whereas activation of the latter suppresses it (Terrill et al., 2016, 2018; Calderwood et al., 2020). What remains unclear is how targeting these receptors affects LS activity. At the circuit level, projection-specific manipulations that involve the upstream hippocampus and downstream hypothalamus provide further information about LS connections involved in food intake. First, optogenetic activation and inhibition of the dCA3/hilus→dLS/MS pathway reduces and increases food intake, respectively (Azevedo et al., 2019). Second, optogenetic activation of the vCA3→vl-LS pathway reduces food intake (Sweeney and Yang, 2015). Third, optogenetic activation and inhibition of the paraventricular nucleus of the hypothalamus (PVN)→vLS pathway reduces and increases food intake, respectively (Xu et al., 2019). These results indicate a suppressive role of both dLS and vLS subregions and their upstream connections in food intake. Downstream of the LS, optogenetic activation of the dLS→LHA pathway also reduces food intake (Sweeney and Yang, 2016; Azevedo et al., 2020; Mu et al., 2020). Together, at least four LS-associated pathways, namely, the dCA3→dl-LS, vCA3→vl-LS, PVN→vLS, and dLS→LHA pathways, appear to be exclusively involved in suppression of food intake. Additionally, the regulation of food intake can also be achieved through modification of taste preference, as optogenetic inhibition of the LHA→LS pathway enhances sweet taste preference (Fu et al., 2019). One potential concern is that the reduction on food intake could be confounded by an upregulation of negative affects (Xu et al., 2019). However, ample evidence argues against this possibility: pharmacological activation of LS GLP-1 receptors affects food intake but not anxiety (Terrill et al., 2016); similarly, chemogenetic activation of LS vGAT or NTS neurons affects food intake but not anxiety (Sweeney and Yang, 2016; Azevedo et al., 2020). These results suggest that the LS’s role in suppressing food intake is largely independent of promoting negative affects. On the other hand, a dissociable role of the LS in regulating negative affects including anxiety and stress has been well studied and is discussed below.

## LS-Associated Circuitry in Anxiety

Mounting evidence also supports a role of the LS in anxiety and stress. To characterize anxiety-like behaviors in rodents, a battery of behavioral paradigms has been developed, including but not limited to open field (OF), light-dark box (LD), elevated plus maze (EPM), novel object, and shock probe tests. In these tests, a decreased time exploring the center area of the OF, the light compartment of the LD, open arms of the EPM, novel objects, or an increased time burying the shock probe, is often considered to be associated with anxiety and stress. Utilization of these paradigms has revealed subregional and molecular profile differences within the LS that allows it to be capable of both promoting and suppressing anxiety-like behaviors.

### LS and anxiety promotion

A variety of stressors can induce LS neuronal activation, evidenced by increased immediate early gene expression (Sheehan et al., 2004). Electrophysiology results provide further evidence that most dLS neurons increase activity under anxiogenic conditions on exploration of the open arms of an EPM relative to the enclosed arms (Thomas et al., 2013). Additionally, optogenetic activation and inhibition of a subset of lateral LS neurons (that express CRF2 receptors) promotes and suppresses multiple anxiety-like behaviors, respectively (Anthony et al., 2014). Projection-specific manipulation indicates that these CRF2 neurons preferentially project to the anterior hypothalamic area (AHA) and promote anxiety via an upregulation of blood corticosterone levels (Anthony et al., 2014). Upstream of the LS, two input regions, namely, the PVN and IL, have been shown to regulate LS activity and promote anxiety-like behaviors as well. Specifically, optogenetic activation of the PVN→vLS pathway promotes stress-related grooming and escape behaviors (Xu et al., 2019), while optogenetic activation of the IL→LS pathway decreases exploration of the center area of the OF or open arms of the EPM (Chen et al., 2021). Together, three LS-associated pathways, namely the PVN→vLS, IL→LS, and lateral LS (CRF2 neurons)→AHA pathways, have been identified to play a key role in promoting anxiety-like behaviors. On the other hand, the LS has also been implicated in suppressing anxiety-like behaviors, which is discussed below.

### LS and anxiety suppression

In contrast to CRF’s role in promoting anxiety/stress, intra-LS administration of neuropeptide Y decreases anxiety-like behaviors, evidenced by increased social interaction and other active coping behaviors (Kask et al., 2001; Trent and Menard, 2011). Additionally, activation of the LS serotonin 1A receptor reduces stress and promotes active coping behavior during a forced swimming test (Singewald et al., 2011). At the circuit level, available evidence suggests that ventral hippocampal efferents to the LS plays a key role in reducing anxiety-like behaviors. This is evidenced by chemogenetic activation and inhibition of LS-projecting vCA1/vCA3 neurons resulting in a decrease and increase in overall anxiety-like behaviors, respectively (Parfitt et al., 2017). Consistently, the LS-projecting vCA1 neurons are activated when animals battle against anxiogenic conditions, on exploring the open arms of an EPM (Wang et al., 2011; Kosugi et al., 2021). Together, these findings indicate a role of the vCA1→vLS and vCA3→vLS pathways in suppressing anxiety-like behaviors.

These seemingly conflicting roles of the LS in both promoting and suppressing anxiety infer heterogeneity in LS subpopulations. Whether this can be attributed to differences of subregions, neuron types, or pathways remains to be fully elucidated, though available evidence points to the opposite roles of dLS versus vLS in promoting and suppressing anxiety-like behaviors, respectively. A closely related yet distinguishable affect to anxiety is fear, which the LS also has strong ties to. While anxiety is a long-lasting state that persists despite the absence of a specific threat, fear is often elicited by an immediate danger or threat (Davis, 1992; Rosen and Schulkin, 1998).

## LS-Associated Circuitry in Fear

Seminally, damage to the LS resulted in septal rage characterized by increased fear responses to harmless stimuli, which led to the hypothesis that the LS plays an important role in regulating fear (Brady and Nauta, 1953). Follow-up research largely pointed to the LS’s role in suppression or relief of fear (Thomas et al., 1991; Sheehan et al., 2004). However, latest research revealed that the LS is capable of both suppressing and promoting fear responses, depending on subregion differences. Most of the research employed a fear conditioning procedure that required animals to learn the association between a cue/context and a fearful stimulus (e.g., footshock), and freezing response was widely used as an indication of fear.

### LS and fear suppression

While lesion studies often disrupted large portion of the LS, application of more selective techniques, such as optogenetics, have identified a major role of the lateral subregion in suppressing fear. A key finding is that the dl-LS and hippocampal CA3 neurons exhibit highly correlated activation under fearful conditions; in particular, increased dl-LS activity immediately precedes the reduction of freezing responses, indicating a dCA3→dl-LS pathway in the suppression of fear (Besnard et al., 2019). Similarly, optogenetic activation of vCA3 terminals in the vl-LS also suppresses freezing responses on exposure to a fearful context (Besnard et al., 2020). Further *in vivo* calcium imaging work identified a subpopulation of LS SST-positive neurons whose activity precedes the reduction of freezing responses (Besnard et al., 2019). Together, these results provide compelling evidence that both the dCA3→dl-LS and vCA3→vl-LS pathways, which likely recruit SST-positive LS neurons, play a key role in the suppression of fear.

### LS and fear promotion

In opposition to the role of the dCA3/vCA3 associated LS pathways in suppressing fear, contrasting studies point to the role of the dCA1→dm-LS pathway in promoting fear. First, transection of the CA1/Sub projection to the LS to anatomically disrupt this pathway abolishes fear responses on exposure to a fearful context or cue (Hunsaker et al., 2009). Second, optogenetic inhibition of the dCA1/Sub→dm-LS pathway decreases freezing responses on exposure to a fearful context (Opalka and Wang, 2020). These results provide initial evidence of the role of the dCA1/Sub→dm-LS pathway in promoting fear, though more evidence is needed to confirm this notion. On the other hand, whether the vCA1→vm-LS pathway suppresses fear remains to be validated. Nonetheless, the above findings update the original hypothesis of the LS’s role in fear suppression by extending to include fear promotion as well.

To date, there is a lack of understanding of how the LS regulates physiological conditions induced by fear, whereas it has been shown that the LS’s involvement in anxiety can be mediated by increased blood corticosterone levels (Anthony et al., 2014). Through its dense projections to various hypothalamic nuclei, it is plausible that the LS regulates increases in stress hormones and blood pressure that manifests into fear responses. On the other hand, most studies on LS use similar foot shock paradigms and freezing as a measure of fear. While this is a well-established method, it does limit the interpretations of the results to this specific type of associative fear. In fact, classical LS lesion studies also reported other fear related behavioral changes such as fear aggression (Brady and Nauta, 1953).

## LS-Associated Circuitry in Social Behavior

Indicative of the LS having a role in social behaviors, it is enriched with receptors for two neuropeptides, vasopressin and oxytocin, both of which are important modulators of social behaviors (Donaldson and Young, 2008). Emerging evidence suggests that the LS plays a key role in a variety of social behaviors, including social aggression (e.g., attack, dominance), social fear, mate bonding, family bonding, and social play. The LS circuitry associated with aggression has begun to be revealed; however, the LS-associated circuitry in other social behaviors remains largely unstudied.

### LS and social aggression

Septal rage animals exhibit exaggerated defensive aggression toward conspecifics, indicating a role of the LS in social aggression (Sheehan et al., 2004). Recent studies provide further evidence in dissecting the downstream and upstream connections of the LS in mediating social aggression. Downstream of the LS, the ventromedial hypothalamus (VMH) is established as the center for eliciting social aggression, evidenced by an immediate onset of attack behavior against an intruder in direct response to optogenetic activation of VMH neurons (Lin et al., 2011). Expectedly, optogenetic activation and inhibition of the lateral LS→VMH pathway, which inhibits and activates the VMH, decreases and increases social aggression, respectively (Wong et al., 2016). Upstream of the LS, two hippocampal subregions, namely, the CA2 and vCA1, are known for their social functions (Hitti and Siegelbaum, 2014; Okuyama et al., 2016). Building off this information to include pathway specificity, optogenetic activation of the CA2→lateral LS pathway, which ultimately activates the VMH via a dLS→vLS disinhibition mechanism, promotes social aggression (Leroy et al., 2018). Together, these findings indicate a key role of the CA2→dLS→vLS→VMH pathway in promoting social aggression. It should be noted that this conclusion derives from studies exclusively on male rodents; paradoxically, in female rats, social aggression is associated with activation of the vLS and suppression of the dLS (Oliveira et al., 2021). This opposite role of the dLS versus vLS in male and female social aggression remains to be reconciled and thus needs further investigation.

### LS and other social behaviors

In addition to aggression, another conflictive social behavior is social fear. One characterization of social fear is by pairing an investigation of a conspecific with a footshock. Using such paradigm, it was found that intra-LS administration of oxytocin increased the amount of time spent investigating a social conspecific despite being previously associated with a footshock (Zoicas et al., 2014). This indicates a critical role of LS oxytocin in the suppression of social fear.

Social behaviors can also be cooperative when there is a beneficial interaction between conspecifics. Using such paradigms, lesioning of the LS disrupted kinship behavior and family bonding, indicating a key role of the LS in social bonding (Clemens et al., 2020). Related to neuropeptides, intra-LS administration of vasopressin in male prairie voles facilitates mate bonding, whereas blocking vasopressin receptors eliminates this behavior (Liu et al., 2001). Manipulation of the vasopressin and oxytocin systems in the LS also modulates social play and social approach (Bredewold et al., 2014; Kelly et al., 2020). Together, these findings indicate an important role of the LS in mate bonding, family bonding, social play, and social fear. The neural pathways that support these social behaviors are unclear; however, the LS likely receives oxytocin and vasopressin from the PVN and bed nucleus of the stria terminalis (BNST), respectively, in executing these social functions (De Vries and Buijs, 1983).

## LS-Associated Circuitry in Memory

Given the critical role of the LS in fear and social experiences, it is conceivable that the LS also engages in associated fear and social memory processes. However, despite being a major target of hippocampal efferents, the LS has not been well established for a potential role in memory processes. Until quite recently, evidence that equivalently supports this notion started to emerge. Overall, the LS has been shown to play an important role in each of the memory stages, including memory acquisition (encoding), memory consolidation (transformation of recent memory into long-term memory), and memory retrieval/expression. Most of the relevant studies use a fear conditioning or social investigation paradigm, which will be the focus of the following discussion.

### LS and fear memory

Growing evidence supports the role of the LS in fear memory processes. First, the LS is critical for fear memory acquisition, as disruption of LS activity during fear conditioning training impairs subsequent memory performance, evidenced by reduced freezing responses on re-exposure to footshock chambers (Desmedt et al., 1999; Calandreau et al., 2007, 2010; Hunsaker et al., 2009; Opalka and Wang, 2020). Notably, this role of the LS in acquisition appears to be dependent on its input from the hippocampus (Hunsaker et al., 2009; Opalka and Wang, 2020). Second, the LS is critical for fear memory consolidation, as a post-training manipulation of the LS (intra-LS administration of CRF) immediately after an inhibitory avoidance task enhances relevant fear memory (Lee, 1995); however, more studies are needed to confirm this notion. Third, the LS is critical for fear memory retrieval/expression, as disruption of LS inputs from the hippocampus during fear memory retrieval impairs memory performance (Hunsaker et al., 2009; Besnard et al., 2019, 2020; Opalka and Wang, 2020). One potential concern of this finding is locomotor effects, which confounds interpretation of fear memory that often utilizes freezing behavior as a readout. However, none of these studies found significant changes in locomotion on manipulation of hippocampal efferents to the LS, which is also consistent with the notion of its lack of role in initiating locomotion or running (Bender et al., 2015). Together, these results provide compelling evidence that the LS plays a critical role in fear memory acquisition, consolidation, and likely retrieval/expression.

### LS and social memory

Another line of evidence that supports the role of LS in memory processes is derived from studies on social behavior. A post-training intra-LS administration of either vasopression or oxytocin immediately after a social interaction experience enhances relevant social memory (Dantzer et al., 1988; Popik et al., 1992). Conversely, a post-training intra-LS administration of the vasopression or oxytocin receptor blocker impairs relevant social memory (Dantzer et al., 1988; Everts and Koolhaas, 1997; Lukas et al., 2013). These results provide compelling evidence of the LS’s role in social memory consolidation. On the other hand, whether the LS is involved in social memory acquisition remains unclear, as the available, yet limited, evidence is mixed. One study reported that intra-LS administration of a vasopressin or oxytocin receptor blocker immediately before a social interaction impairs the pair bond formation (Liu et al., 2001). In contrast, another study reported that intra-LS administration of an oxytocin receptor blocker does not affect social memory formation (Lukas et al., 2013). Lastly, whether the LS is involved in social memory retrieval/expression has not been investigated.

### LS and spatial navigation

Spatial navigation is dependent on the hippocampus and thus widely used in memory related studies. In particular, hippocampal place cells that encode specific spatial positions are believed to form a cognitive map of the environment (O’Keefe and Nadel, 1978). Like hippocampal place cells, a subset of LS neurons, albeit in a smaller percentage, have been shown to exhibit place coding properties (Zhou et al., 1999; Leutgeb and Mizumori, 2002; Takamura et al., 2006; Wirtshafter and Wilson, 2020; van der Veldt et al., 2021). In general, LS place cells are described as of lesser quality than hippocampal place cells, often with larger or non-focalized place fields. However, not all studies have successfully identified place cells in the LS, which may be because of recording from different LS subregions (Tingley and Buzsáki, 2018). Rather, some LS neurons transform hippocampal spatial information via a phase-coding strategy, that is, these neurons code place fields by firing at specific phases of hippocampal theta oscillation (Tingley and Buzsáki, 2018). In addition, LS neurons also appear to support spatial navigation by incorporating self-motion information such as speed, acceleration, and direction (Wirtshafter and Wilson, 2020; van der Veldt et al., 2021). Together, these studies provide considerable amount of evidence in supporting the LS’s role in spatial information coding and likely memory-guided spatial navigation.

### LS and sharp-wave ripple

Latest research revealed that the LS displays a fast oscillation, similar to the ∼200-Hz hippocampal ripple oscillation that occurs predominantly during slow-wave sleep and plays a key role in memory consolidation (Buzsáki, 2015). Importantly, this LS fast oscillation is temporally locked to hippocampal ripple, indicating an information flow that conveys ripple-associated memory information from the hippocampus to LS (Tingley and Buzsáki, 2020). Moreover, many LS neurons display a postripple activation that correlates with ripple amplitude (Wirtshafter and Wilson, 2019; Tingley and Buzsáki, 2020), further supporting a hippocampus→LS routing of information flow. Interestingly, hippocampal ripple oscillation appears to control the LS for parrel regulation of whole-body glucose homeostasis and memory consolidation (Tingley et al., 2021). This line of evidence provides initial insight into the neural mechanism of hippocampus→dLS communication for memory consolidation.

### LS-associated memory circuitry

Few studies have examined the LS-associated circuitry in memory processes; however, inputs from hippocampal CA3, CA2, CA1 and subiculum, and outputs to hypothalamic and mammillary areas are likely the major connections (Risold and Swanson, 1997b). Indeed, the dCA1→dm-LS and dCA3→dl-LS pathways have been shown to be involved in fear memory acquisition and discrimination processes, respectively (Besnard et al., 2020; Opalka and Wang, 2020). Previous research largely agree on a hippocampal–neocortical communication in supporting cognitive aspects of memory functions (Shohamy and Turk-Browne, 2013). We propose that the hippocampus→LS communication accounts for non-cognitive aspects of memory functions that mediate physiological changes, such as stress hormones and blood pressure to prepare the animal for a predicted immediate danger. These non-cognitive responses may be mediated by a few LS downstream regions including the hypothalamus and mammillary areas (Roy et al., 2017).

## Conclusions

**Figure 3.**
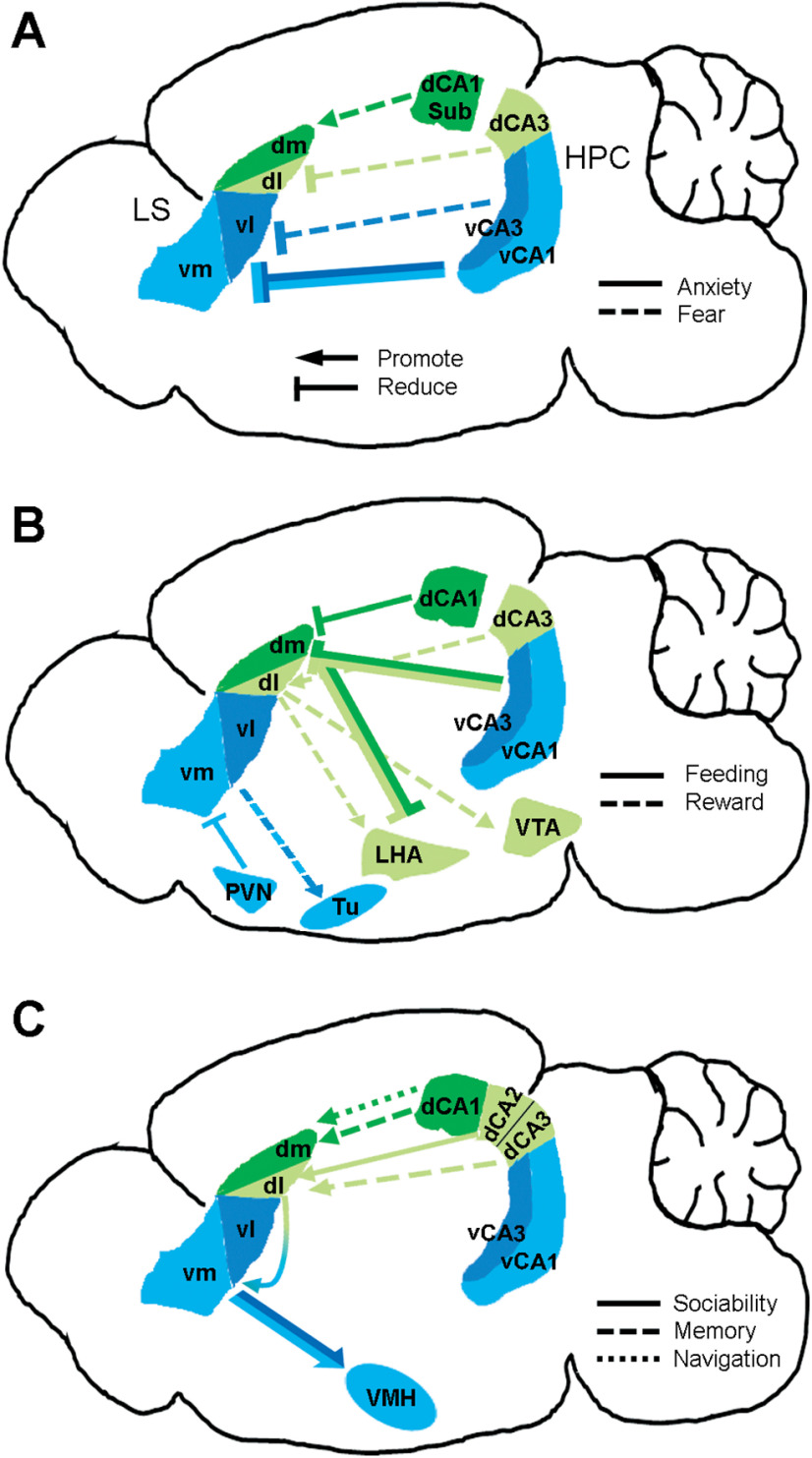
LS subregion associated neural circuits and functions. ***A–C***, Major LS subregion connections in fear/anxiety (***A***), reward/feeding (***B***), and other functions (***C***). Note: results from studies that do not differentiate LS subregions are not shown. LS, lateral septum; LHA, lateral hypothalamic area; HPC, hippocampus; PVN, paraventricular nucleus of the hypothalamus; Sub, subiculum; Tu, hypothalamic tuberal nucleus; VMH, ventromedial hypothalamus; VTA, ventral tegmental area.

In the current review, we consolidated the multifaceted functions of the LS in reward, feeding, anxiety, fear, sociability, spatial navigation, and memory (for other functions such as thermoregulation, cardioregulation, and pain, please see Sheehan et al., 2004). These functions have been mapped in association with distinct LS subregion connections and are summarized below (Fig. 3). This is an exhaustive list of LS functions, but each is not mutually exclusive (Table 1). While each pathway has its own function, multiple pathways could be activated at any given time. These parallel pathways sometimes antagonize each other via the reciprocal GABAergic connections between LS subregions (Sheehan et al., 2004; Leroy et al., 2018; Oliveira et al., 2021). Overall, the multifaceted functions of the LS seem to have a commonality in that they are related to the context or environment of the animal. This often has to do with the salience of a stimulus or other environmental changes, being either positive or negative valence. We propose that the LS as a whole could influence behavior by continuously combining and integrating contextual information from all LS subregions. This communication between LS subregions is likely essential for flexible assigning valence to contextual elements, which need constant updating in an ever-changing environment for animals to make appropriate responses.

**Table 1:** Major LS-associated neural circuitry and functions

	Reward	Feeding	Anxiety	Fear	Sociability	Memory
dCA1/Sub→dm-LS				**+**		**+**
CA2→dLS→vLS→VMH (males only)					***–***	
dCA3→dl-LS	**+**	***–***		***–***		**+**
vCA1/CA3→vLS			***–***			
vCA3→vl-LS		***–***		***–***		
IL→LS		**+**	**+**			
dl-LS→VTA	**+**					
dLS→LHA	**+**	***–***				
LS (CRF2)→AHA			**+**			
vLS→Tu	**+**					
PVN→vLS		***–***	**+**			

**+**, promotion effect; –, suppression effect; AHA, anterior hypothalamic area; IL, infralimbic cortex; LHA, lateral hypothalamic area; PVN, paraventricular nucleus of the hypothalamus; Tu, hypothalamic tuberal nucleus; VMH, ventromedial hypothalamus; VTA, ventral tegmental area.

### LS subregions and major functions

Previous studies have delineated the LS into subregions mainly based on cytoarchitectural factors and/or molecular markers that do not specifically relate to its connectivity (Risold and Swanson, 1997b; Sheehan et al., 2004). While those previous subregional insights are important for understanding the LS, our proposed classification of LS subregions gives rise to functionally relevant and more clear pathway delineations involving the LS. Specifically, we characterized the LS into four major subregions, namely, dl-LS, dm-LS, vl-LS, and vm-LS, solely based on its major efferent and afferent connections that are also translated to distinct functions.

In the dorsal dimension, the dl-LS has been well studied for its role in reward and drug abuse. Manipulation of this subregion influences VTA dopamine activity, thus regulating various reward-seeking and drug-seeking behaviors (Luo et al., 2011; Jiang et al., 2018; McGlinchey and Aston-Jones, 2018; Werner et al., 2020). Moreover, this promotion of reward is in line with another role of the dl-LS in suppressing negative affect such as fear (Besnard et al., 2019). On the other hand, the dm-LS is less well studied. Available evidence suggests that the dm-LS and dl-LS exhibit opposing functions in promoting and suppressing learned fear, respectively (Hunsaker et al., 2009; Besnard et al., 2019; Opalka and Wang, 2020). This difference could be explained by the finding that the dm-LS is predominantly involved in memory processes (via its inputs from the dCA1/Sub), whereas the dl-LS is involved in both memory (via its inputs from the dCA3) and affective processes (via its output to VTA), with the affect component often masking the memory component. Additionally, studies that target either the dl-LS or entire dLS exhibit similar effects in suppressing feeding (Sweeney and Yang, 2016; Azevedo et al., 2020; Mu et al., 2020).

In the ventral dimension, the vl-LS and vm-LS exhibit similar functions. First, activation of either or both subregions reduces fear and anxiety (Parfitt et al., 2017; Besnard et al., 2020). Second, activation of either or both subregions reduces social aggression (Leroy et al., 2018). Third, activation of either or both subregions reduces feeding (Sweeney and Yang, 2015; Xu et al., 2019). These findings indicate a general role of the vLS in suppressing negative affect, including fear, anxiety, aggression, and those associated with hunger state. Moreover, this suppression of negative affect is in line with another role of the vLS in promoting reward (Mu et al., 2020).

Collectively, three LS subregions, namely, the dl-LS, vl-LS, and vm-LS subregions, exhibit largely similar functions in promoting reward while suppressing negative affect. However, there seem to be differences: while the dl-LS directly, the vl-LS and vm-LS indirectly promotes reward (that is, anti-fear or anti-anxiety). Conversely, while the vl-LS and vm-LS directly, the dl-LS indirectly suppresses negative affect (that is, pro-reward). Additionally, the similar roles of the three subregions in suppressing feeding could be explained by that the vl-LS and vm-LS directly suppress hunger state, whereas the dl-LS promotes reward and thus, may indirectly alleviate hunger. On the other hand, the dm-LS exhibits opposite functions compared with the above three subregions, which could be explained by its major role in supporting memory processing rather than regulating affect. Nonetheless, the functional differentiation of LS subregions has yet to be fully revealed, as the regions needs to be more closely studied.

### Outlook

Recent studies have provided an important division between LS subregions; however, further circuit mapping using targeted techniques are required to fully determine the roles of each subregion in the discussed functions as well as determine whether specific projections involve distinct or overlapping populations of LS neurons. It has also become increasingly clear that the LS is not simply a relay structure; rather, it actively integrates hippocampal and subcortical information, often taking advantage of subregion coordination within the LS, before passing on to downstream regions. Future work that employs neuronal recording techniques such as *in vivo* electrophysiology and calcium imaging are particularly necessary to shed light on how individual LS neurons and their communications within and outside of the LS contribute to emotion, cognition, behavior, and action selection. Additionally, further work that investigates LS subregional connectivity and interactions should provide new and important insights into the functional heterogeneity as well as integrity of the LS in regulation of effect and behavioral outputs.

## Synthesis

Reviewing Editor: Alexxai Kravitz, Washington University in St. Louis Decisions are customarily a result of the Reviewing Editor and the peer reviewers coming together and discussing their recommendations until a consensus is reached. When revisions are invited, a fact-based synthesis statement explaining their decision and outlining what is needed to prepare a revision will be listed below. The following reviewer(s) agreed to reveal their identity: NONE.

Thanks to the authors for being responsive to all prior critiques, and congrats on an excellent review!

## Author Response

Synthesis Statement for Author (Required):

Your manuscript was seen and discussed by two reviewers and myself, and we all had a similar set of conclusions about it. To orient you to what we discussed, we all thought the review topic was timely and important, and contained a thorough compilation of relevant literature on the lateral septum. That said, there were several aspects that were lacking and could be improved.

RESPONSE:

We would like to thank Dr. Kravitz and the reviewers for their constructive and helpful comments. Accordingly, we have revised our manuscripts to address those concerns.

The critical points we discussed were:

1) The review covers a lot of ground but offers little critical synthesis. It would be helpful to include a broader model, or the authors’ opinions on what the lateral septum is doing (is it doing everything?). The review should also better illuminate outstanding questions and experiments for future study.

RESPONSE:

We appreciate this critique. During our revision, we synthesized important findings and included a broader model in the Conclusion section (Lines 422-437). We also identified less-studied and outstanding questions, both towards the end of each major section and in the Conclusion section (Lines 477-489).

2) The writing is at times list-like, and would greatly improve if the authors provided more context for how the cited papers came to various conclusions. Rather than listing through references, it would be helpful to add a sentence explaining what critical insight was contributed from each paper, and how the researchers came to that insight.

RESPONSE:

We thank the editor and reviewers for pointing out this weakness, and we agree that specific experimental details and context were unintentionally left out and should have been included in our discussions. Accordingly, we have largely revised our manuscript to mitigate such weakness. Throughout the revision, we have included more detailed explanations and transitions between each of the subsections to connect each of the findings. It is now a logical flow that has directionality of how each function is related to the one subsequent to it. This has elevated our implications and lead to more accurate depictions of these studies.

3) Reviewer 1 and I were also both confused by the placement of the section on anatomy that came after the “Conclusions” section. It almost seems like this section was moved in the draft and was meant to be somewhere else? Please confirm, and consider integrating this section better into the main text. Having it appear after “Conclusions” threw me for a loop.

RESPONSE:

We appreciate the advice. Accordingly, we have replaced the list with a table (Table 1) as suggested by reviewer 1.

4) There was a lack of clarity around terminology in multiple areas, such as anxiety, stress, and food approach. See comments from reviewer 2.

RESPONSE:

We have clarified the definition of the terminology (see our reply to reviewer 2).

Reviewer 1

The authors present a useful overview of recent papers studying the role of LS in behavior. This review is timely and will be of value to the field. However, I have some concerns about the structure of the article. Primarily, since the authors cover such a broad range of behaviors, it is sometimes difficult to understand how all of these findings relate to one another. Below are my specific recommendations:

1) Lines 360-388: I’m not sure why the authors have a brief conclusion paragraph here followed by a list of circuits relevant to different behaviors. I would consider making this into a table that lists the recent circuitry related to LS and the behaviors involved. This would be really helpful and should improve the visibility of the manuscript.

RESPONSE:

We thank the reviewer for this constructive suggestion. Accordingly, we have replaced the list with a table (Table 1) as recommended.

2) The review would be improved by increased synthesis between the different sections. At times, the article reads as a list of different/seemingly unrelated findings on the function of LS, and it is difficult to understand how these findings relate to one another.

RESPONSE:

Throughout the revision, we have included more detailed explanations and transitions between each of the subsections to connect each of the findings. It is now a logical flow that has directionality of how each function is related to the one subsequent to it. This has elevated our implications and lead to more accurate depictions of these studies.

3) Lines 390-459: It would be great if this section was integrated with the previous sections on the different functions of LS so that the reader can begin to understand how these diverse functions of LS may occur, and how they relate to one another.

RESPONSE:

We have taken this consideration into account and have added some of this information to the previous sections so that they can be better integrated with one another. This information in the previous section integrates the different functions based on affect to better connect how the LS is able to play a role in all of these diverse functions. We also mention subregion differences based on the pathway implicated. However, because we are synthesizing a large amount of information, we kept our main conclusions about subregion differences in this conclusion section as a wholistic view of how all of these functions come together in a single region.

4) How was figure 1 generated? Was this taken directly from Allen brain atlas? This is not completely clear in the text.

RESPONSE:

We thank the reviewer for raising this issue. We now specify that all brain section photos are adapted from Allen Brain Atlas - Mouse Connectivity (see Lines 720-721).

Reviewer 2

This review paper on the role of the lateral septum (LS) in a variety of behaviors focuses on anatomical subdivisions with differing connectivity, and how these subregions serve different functions. This is an interesting topic and would be a contribution to the literature. The focus on anatomic connectivity as the defining factor for subregion classification, and the argument that this explains the diversity of effects that can be obtained from LS manipulations, is a novel perspective. The focus on behavior, emotion and memory is also a strength in that reviews covering other physiological functions of this brain area are already available, as the authors noted. The manuscript is well-organized, and the figures are each informative and useful.

Despite these strengths, there are a number of ways in which this manuscript could be improved, outlined below.

Major comments:

1) Throughout much of the review, from sections “LS associated circuitry in reward” through to the “Conclusions,” there is little explanation of critical analysis. Rather, much of the paper reads like a laundry list of conclusions, rather than a true review of the literature. There is a great deal of information to cover here, and I appreciate that the authors may have erred on the side of being too concise in order to limit length, however, one statement after another with no discussion of how one would come to such conclusions - what methods were used, what behavioral task, what was actually measured, etc. - is not really informative. To clarify this point using an example, this sentence from the section on LS and drug abuse: “On the other hand, inhibition of the LS reduces motivation to indulge in drug-seeking behavior.” would leave anyone who hasn’t read the cited publication wondering what exactly “inhibition of the LS” means (chemogenetic, optogenetic, GABA agonist, some other approach?), how motivation and drug-seeking were operationally measured, and what drug(s) was examined here. This problem is compounded for statements about multi-synaptic pathways, where no context is given for how these circuits were implicated.

RESPONSE:

We thank the reviewer for pointing out this weakness, and we agree that specific experimental details were unintentionally left out and should have been included in our discussions. Accordingly, we have largely revised our manuscript to mitigate such weakness.

Throughout the revision, we have included more detailed explanations and transitions between each of the subsections to connect each of the findings. It is now a logical flow that has directionality of how each function is related to the one subsequent to it. This has elevated our implications and lead to more accurate depictions of these studies.

2) The section on anxiety suffers from conflation of stress and anxiety, and in some cases anthropomorphizing rodent experimental subjects. The behavioral tasks used can assess what some researchers call “anxiety-like behavior” but it is not possible to conclude that manipulations increase or decrease anxiety in rodents because anxiety is a human construct. See guidance from NIMH on this issue: https://www.nimh.nih.gov/about/director/messages/2019/what-can-animals-tell-us-about-mental-illnesses

RESPONSE:

We have made the appropriate changes in both explaining how these methods best attempt to study anxiety in rodents and changing the language to “anxiety-like behavior” to correct the anthropomorphizing of the experimental subjects (see Lines 206-214).

3) Potential confounding factors are mentioned in a few sections, but there is no serious attention given to these, and these interpretive issues are not well-integrated into the discussion. For example, in the section on LS involvement in food intake, it is mentioned that food intake suppression could be explained by “increased anxiety, stress, avoidance or escape behavior” and this is possible, but if that is the case, then the results summarized in this paragraph do not provide compelling evidence for a role of the LS in feeding. There are several papers that have examined a role of the LS in the interaction between stress and feeding behavior (e.g., does LS circuitry mediate stress-induced hypophagia?), and there are findings relating to LS GLP-1 receptors in particular that support independence of these functions. None of this is discussed here. Another example of this is in the LS and fear memory section, where it is noted that manipulation of the LS or inputs from hippocampus may cause locomotor changes. Locomotor effects would be a serious interpretive problem for all behavioral tasks that rely on movement, which include not only fear conditioning paradigms but also just about every other behavioral task mentioned throughout this review paper. Immediately after noting this confound, the authors state that there is compelling evidence that the LS plays a critical role in fear memory, but there is no explanation for why they feel that the confounding motor issues can be so readily dismissed here.

RESPONSE:

We thank the reviewer for raising this issue.

In the feeding section, we have clarified our interpretation of these confounding factors. While there is one single study reporting LS manipulation on changes of both feeding and negative affects, most other studies found dissociated function in feeding from anxiety. Therefore, we have revised our previous conclusion by stating that the LS’s role in suppressing food intake is largely independent of promoting negative affects (Lines 197-205).

In the memory section, this mention of locomotor changes as a confounding variable was revised because none of the studies found significant locomotor changes during the tasks (Lines 359-365). Separately, the LS’s involvement in locomotion is discussed in the Spatial Navigation section (Lines 380-396).

Minor comments:

1) Food approach is not synonymous with motivation, and operant responding for food is not necessarily a measure of food approach. In some cases, the authors explained concepts very nicely (e.g., distinguishing fear from anxiety), but this is one example where more explicit definition would be particularly helpful.

RESPONSE:

We agree that food approach is not synonymous with motivation. To clarify this, we now only use “food motivation” throughout in our revised manuscript. We also added an explanation that, by food motivation we are insinuating the animals are seeking food through operant responding, such as nose poke or lever press (Lines 159-163).

2) Many sentences throughout the paper contain odd phrasing or are so long and complicated that while grammatically correct, they can be difficult to understand without reading several times. Editing with an eye toward readability would be useful here.

RESPONSE:

We have read through and either edited sentences that appeared to have odd phrasing or added additional information for clarification for the reader. In addition, we have sought outside help for editing of our manuscript.
